# Ablation of Prion Protein in Wild Type Human Amyloid Precursor Protein (APP) Transgenic Mice Does Not Alter The Proteolysis of APP, Levels of Amyloid-β or Pathologic Phenotype

**DOI:** 10.1371/journal.pone.0159119

**Published:** 2016-07-22

**Authors:** Isobel J. Whitehouse, Deborah Brown, Herbert Baybutt, Abigail B. Diack, Katherine A. B. Kellett, Pedro Piccardo, Jean C. Manson, Nigel M. Hooper

**Affiliations:** 1 School of Molecular and Cellular Biology, Faculty of Biological Sciences, University of Leeds, Leeds, United Kingdom; 2 The Roslin Institute and Royal (Dick) School of Veterinary Studies, University of Edinburgh, Roslin, Midlothian, United Kingdom; 3 Institute of Brain, Behaviour and Mental Health, Faculty of Medical and Human Sciences, University of Manchester, Manchester, United Kingdom; IRCCS - Mario Negri Institute for Pharmacological Research, ITALY

## Abstract

The cellular prion protein (PrP^C^) has been proposed to play an important role in the pathogenesis of Alzheimer’s disease. In cellular models PrP^C^ inhibited the action of the β-secretase BACE1 on wild type amyloid precursor protein resulting in a reduction in amyloid-β (Aβ) peptides. Here we have assessed the effect of genetic ablation of PrP^C^ in transgenic mice expressing human wild type amyloid precursor protein (line I5). Deletion of PrP^C^ had no effect on the α- and β-secretase proteolysis of the amyloid precursor protein (APP) nor on the amount of Aβ38, Aβ40 or Aβ42 in the brains of the mice. In addition, ablation of PrP^C^ did not alter Aβ deposition or histopathology phenotype in this transgenic model. Thus using this transgenic model we could not provide evidence to support the hypothesis that PrP^C^ regulates Aβ production.

## Introduction

Alzheimer’s disease (AD) is the most common form of dementia affecting 30 million individuals world-wide [1,2]. Age is the greatest risk factor for AD, with the incidence doubling every 5 years after age 65. Therefore, with our ageing population, AD is placing immense financial and social pressure on society. Currently there are no treatments that either cure or halt the progression of this neurodegenerative disease [3]. The majority (>95%) of AD cases have no underlying genetic mutation and are referred to as sporadic or late-onset AD [4]. In a small proportion of cases, mutations in the genes encoding the amyloid precursor protein (APP) or presenilin (PS) 1 or PS2 give rise to early onset, familial AD [4]. The disease is characterised by the deposition in the brain of extracellular plaques of amyloid-β (Aβ) which is derived from the proteolytic processing of APP [5], along with intracellular neurofibrillary tangles of hyperphosphorylated tau protein [6]. APP is cleaved first by the β-secretase, β-site APP cleaving enzyme-1 (BACE1), and then by the PS-containing γ-secretase complex to release Aβ, the predominant forms of which are 40 or 42 amino acids in length (Aβ_40_ and Aβ_42_, respectively) [5].

Cleavage of APP by BACE1 is the rate-limiting step in Aβ production [7] and various cellular proteins have been reported to influence this step, including the cellular form of the prion protein (PrP^C^) [8]. PrP^C^ inhibited the action of BACE1 on wild type APP (APP_WT_) in various cellular models, in part through glycosaminoglycan-mediated interaction at the cell surface and in part through retaining the pro-domain containing form of BACE1 in the early secretory pathway [8,9]. In the brains of PrP^C^ null mice there was a significant increase in the amount of endogenous murine Aβ [8], consistent with PrP^C^ having a role in regulating the production of Aβ from APP *in vivo*. Together with the observation that PrP^C^ was decreased in the brains from sporadic AD individuals and that the amount of PrP^C^ inversely correlated with BACE1 activity, soluble and insoluble Aβ and Braak stage in the human brain [10,11], led us to propose that PrP^C^ may function to protect against AD and that loss of PrP^C^ would lead to the earlier onset of AD [12,13].

The inhibitory effect of PrP^C^ on the BACE1 cleavage of APP was only apparent on APP_WT_ and was lost on APP with the Swedish double point mutation adjacent to the BACE1 cleavage site (APP_Swe_) [9]. APP_Swe_ is subject to BACE1 cleavage in the secretory pathway [14,15] rather than in the endosomal pathway as for APP_WT_ [16,17]. As PrP^C^ interacted directly with the prodomain of the immature Golgi-localised form of BACE1, decreasing the amount of BACE1 at the cell surface and in endosomes, this provided a mechanism to explain the differential inhibitory effect of PrP^C^ towards APP_WT_ and APP_Swe_ [9]. In transgenic mice expressing human APP_Swe.Ind_ we [9] and others [18–20] have reported that upon genetic ablation of PrP^C^ there is no alteration in APP processing, Aβ levels or plaque pathology, consistent with this differential inhibitory mechanism. Therefore, in this study we aimed to determine whether ablation of PrP^C^ in transgenic mice expressing human APP_WT_ results in increased Aβ and subsequently causes the premature appearance of plaque pathology.

## Materials and Methods

### Transgenic Animals

Transgenic APP_WT_ mice over expressing human wild-type APP (line I5) or APP_Swe,Ind_ mice over expressing human APP with the Swedish (K670N/M671L) and Indiana (V717F) familial AD mutations (line J20) [21] were obtained from The Jackson Laboratory, (Line B6.Cg-Tg(PDGFB-APP)5Lms/J, stock number 004662 and B6.Cg-Tg(PDGFB-APP_Swe,Ind_)20Lms/2J, stock number 006293, respectively) and The J. David Gladstone Institutes, San Francisco, CA 94158) and crossed with inbred PrP knockout mice (129Ola PrP^-/-^) [22]. The genetic background of all mice used in this study was mixed B6/129Ola and only female mice were used. Operators were blinded to genotype and animals were randomly assigned to cages of n = 4 and given access to food and water ad libitum. During housing, animals were monitored daily for health status and no adverse effects were noted. All the transgenic mice used in this study were genotyped. DNA was prepared from ear punch tissue using a DNeasy Kit (Qiagen). PCR was performed using the protocol specific for these mice from The Jackson Laboratory. At end point, animals were culled by cervical dislocation and brain hemispheres were either frozen at -80°C for biochemical analysis or fixed in 10% formol saline for histopathological analysis. These experiments were approved by The Roslin Institute’s Animal Welfare and Ethical Review Board and were conducted according to the regulations of the UK Home Office Animals (Scientific Procedures) Act 1986. All efforts were made to minimise suffering.

### Homogenisation

Brain hemispheres were homogenised using a two-step extraction protocol [23]. Briefly, initial homogenisation (120 mg/ml wet weight) was carried out using an electrical homogeniser in 2% (w/v) SDS containing protease inhibitor cocktail (Roche Diagnostics GMbH, Germany) and PhosSTOP phosphatase inhibitor (Roche Diagnostics GMbH, Germany), followed by centrifugation at 100,000 *g* for 1 h at 4°C. The resultant supernatant (containing ‘soluble’ Aβ) was collected and analysed as described below. The pellet was extracted in 70% (v/v) formic acid in dH_2_O followed by centrifugation at 100,000 *g* for 1 h at 4°C. The supernatant (containing ‘insoluble’ Aβ) was collected and analysed as described below.

### SDS-PAGE and Immunoblot Analysis

Samples were mixed with an equal volume of SDS dissociation buffer (125 mM Tris/HCl, pH 6.8, 2% (w/v) SDS, 20% (v/v) glycerol, 100 mM dithiothreitol, 0.002% (w/v) bromophenol blue) and boiled for 5 min. Proteins from mouse brain homogenate (30 μg) were resolved by SDS-PAGE using 7–17% polyacrylamide gradient gels. Resolved proteins were then transferred to Immobilon P polyvinylidene difluoride membrane (Amersham, Little Chalfont, UK). The membrane was blocked by incubation for 1 h with PBS containing 0.1% (v/v) Tween-20 and 5% (w/v) dried milk powder. Antibody incubations were performed in PBS-Tween containing 2% (w/v) BSA. Antibody 6D11 (Eurogentec Ltd., Liege, Belgium) recognises PrP^C^, antibody Y188 (Abcam, Cambridge, UK) was used to detect full length APP and antibody AC15 (Sigma, Dorset, UK) was used to detect actin. Horseradish peroxidase (HRP)-conjugated secondary antibodies were used in the same buffer. Bound antibody was detected using the enhanced chemiluminescence detection method (Amersham Biosciences, Amersham, UK).

### Measurement of Aβ and Soluble APP Fragments by Mesoscale Discovery Analysis

Biochemical analysis was performed on APP_WT_/PrP^+/+^ (n = 3), APP_WT_/PrP^-/-^ (n = 6) mice at 32 and 75 weeks of age, and APP_Swe,Ind_/PrP^+/+^ (n = 5, 7 and 4 at 5, 10 and 40 weeks, respectively) Aβ peptides (Aβ_38_, Aβ_40_ and Aβ_42_) and soluble APP fragments (sAPPα and sAPPβ) contained in the soluble (SDS extracted) fraction and Aβ peptides in the insoluble (formic acid extracted) fraction were assessed using the Mesoscale Discovery (MSD) platform. Aβ_38_, Aβ_40_ and Aβ_42_ were measured using the V-Plex Aβ peptide panel (6E10) kit and sAPPα and sAPPβ using the sAPPα/sAPPβ multiplex kit according to the manufacturer’s instructions (MSD, Maryland US). SDS samples were diluted 1:40 and formic acid samples 1:500 in diluent 35.

### Histology

Pathological analysis was performed on APP_WT_/PrP^+/+^ (n = 4), APP_WT_/PrP^-/-^ (n = 4) and APP_Swe,Ind_/PrP^+/+^ (n = 4) mice at 32 and 75 weeks of age (the group size dropped to an n = 1 in the 75 week APP_WT_/PrP^+/+^ group due to intercurrent losses). Sections from a 129Ola (WT) mouse at 75 weeks of age were also analysed. Neuropathologic evaluation was performed blind by two operators. Fixed brain tissue was processed and tissue sections were prepared as described [24]. Paraffin sections (6 μm) were stained with hematoxylin-eosin and immunostained with the following antibodies: 4G8 (1/300) raised against Aβ_17–24_ (Covance; SIG-39200); OC (1/500) that recognises a conformational-dependent epitope, specific to fibrils and soluble fibrillar oligomers (a gift from Professor C. Glabe, Dept. Neurology, University of California at Irvine School of Medicine, Irvine, CA, USA); anti-synaptophysin (1/150) that recognises a synaptic vesicle protein (Synaptic systems, 101002); anti-glial fibrillary acidic protein (GFAP) (1/400) (DAKO Z0334); anti-Iba1 (1/1000) a calcium-binding protein specifically expressed in macrophages and microglia (Wako 019–9741). Primary antibody binding was detected with biotinylated goat anti-species specific IgG (Stratech Scientific Ltd.) and the Vectastain Elite ABC Kit (Vector Laboratories), visualized with diaminobenzidine. For synaptophysin immunolabelling antigen retrieval (DAKO S1699) and enhancement visualization system (EnVision^™^ K5007) were used. Antigen retrieval using 0.1M citrate buffer was performed with the Iba1 labelling.

### Statistical Analysis

Densitometric analysis was performed using the advanced image data analyser (AIDA) programme (Raytest Scientific Ltd). The Kolmogorov-Smirnov test was used to determine that the data in each group was normally distributed. Following this the Levene’s test was used to ensure that the data sets were of equal variance. In samples where the data met the criteria of a normal distribution and equal variance, the parametric independent t-test was used to calculate significance. In the samples which were not normally distributed the non-parametric two-tailed Mann-Whitney U test was used to compare two independent samples. The data were analysed using the Statistical Package for Social Sciences (SPSS 19) program (Chicago, USA).

## Results and Discussion

**Fig 1:**
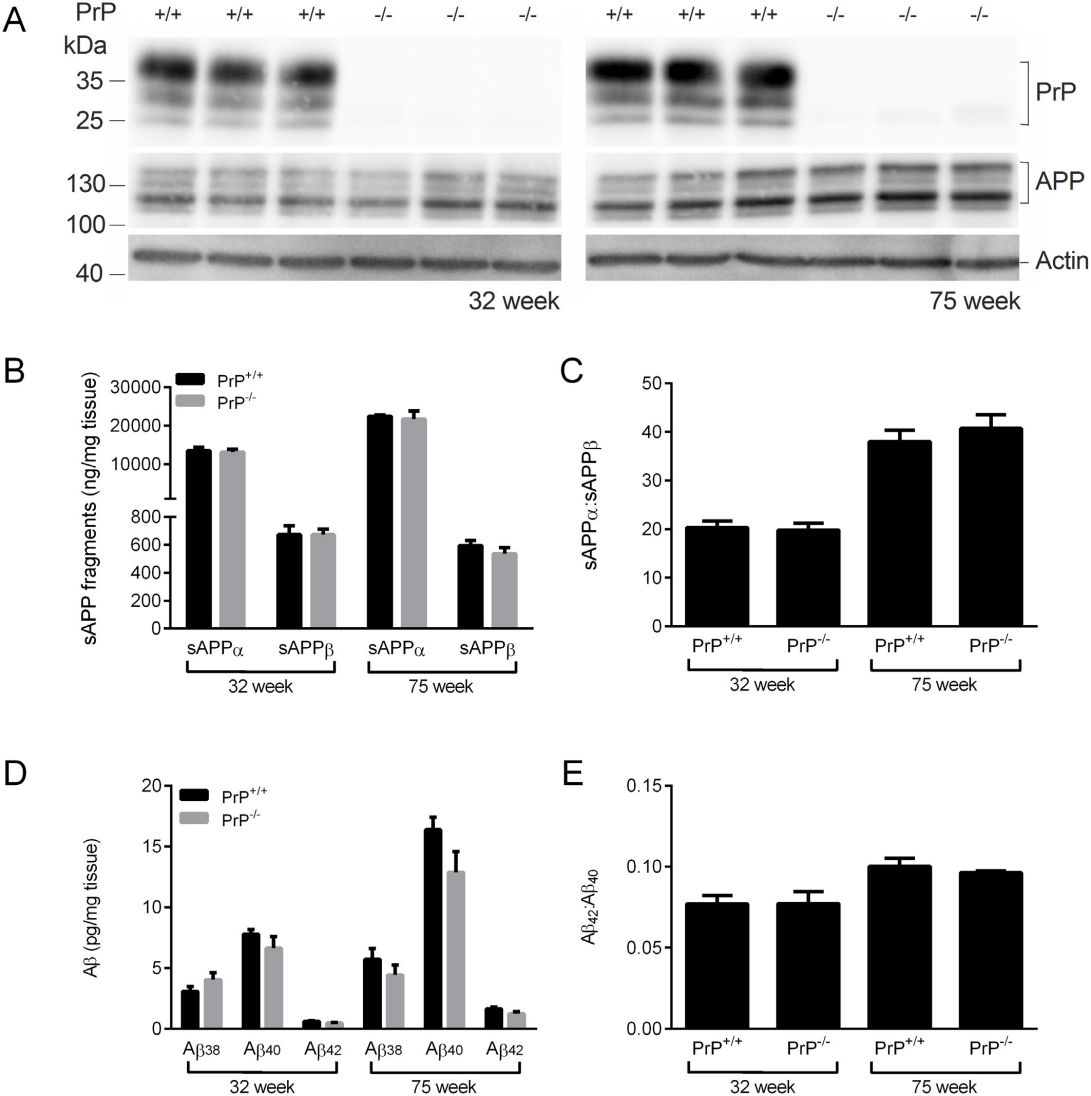
Ablation of PrP^C^ does not affect APP proteolysis or Aβ peptide levels in APP_WT_ mice. Brain hemispheres from 32 week and 75 week old APP_WT_/PrP^+/+^ and APP_WT_/PrP^-/-^ mice were subjected to two-step homogenisation, and the soluble proteins (SDS extracted) were subjected to SDS-PAGE and immunoblotting. (A) representative immunoblots of PrP^C^ and APP, with actin as a loading control. (B) Soluble (SDS extracted) sAPPα and sAPPβ detected by MSD ELISA and (C) the ratio of sAPPα to sAPPβ. (D) Soluble (SDS extracted) Aβ_38,_ Aβ_40_ and Aβ_42_ detected by MSD ELISA and (E) the ratio of Aβ_42_ to Aβ_40_. Data shown as mean ± SEM (n = 3–6). There was no significant difference between the APP_WT_/PrP^+/+^ and APP_WT_/PrP^-/-^ at the same time point for any of the APP or Aβ peptides.

In order to investigate whether ablation of PrP^C^ in transgenic mice expressing human APP_WT_ results in increased Aβ, mice expressing human APP_WT_ [21] were crossed with PrP^C^-null 129/Ola mice [22]. Immunoblot analysis of mice at 32 and 75 weeks of age confirmed the absence of PrP^C^ in the APP_WT_/PrP^-/-^ mice and that the lack of PrP^C^ had no effect on APP expression (Fig 1A). The amounts of the soluble APP species, sAPPα and sAPPβ, were no different between the APP_WT_/PrP^-/-^ and APP_WT_/PrP^+/+^ mice at either 32 or 75 weeks of age (Fig 1B), indicating that deletion of PrP^C^ had no effect on either the α- or β-secretase cleavage of APP. There was a significant increase in sAPPα in both genotypes at 75 weeks of age compared to 32 weeks of age (Fig 1B) which resulted in an increased sAPPα:sAPPβ ratio (Fig 1C). The levels of soluble (SDS-extracted) Aβ_38_, Aβ_40_ and Aβ_42_ were investigated. At both 32 and 75 weeks of age there was no difference in the amount of soluble Aβ_38_, Aβ_40_ or Aβ_42_ in the brain homogenates of the APP_WT_/PrP^-/-^ mice compared to the APP_WT_/PrP^+/+^ mice (Fig 1D). The amount of Aβ_40_ and Aβ_42_ was higher at 75 weeks of age than at 32 weeks of age regardless of *Prnp* genotype (Fig 1D), with a proportionately larger increase in Aβ_42_ such that the Aβ_42_: Aβ_40_ ratio increased in both the APP_WT_/PrP^-/-^ and APP_WT_/PrP^+/+^ mice with age (Fig 1E). The levels of Aβ peptides in the insoluble (formic acid extracted) fraction from either the APP_WT_/PrP^-/-^ or the APP_WT_/PrP^+/+^ mice were below the limit of detection in the assay. These data indicate that lack of PrP^C^ in transgenic mice expressing human APP_WT_ does not alter the proteolysis of APP or the levels of Aβ.

**Fig 2:**
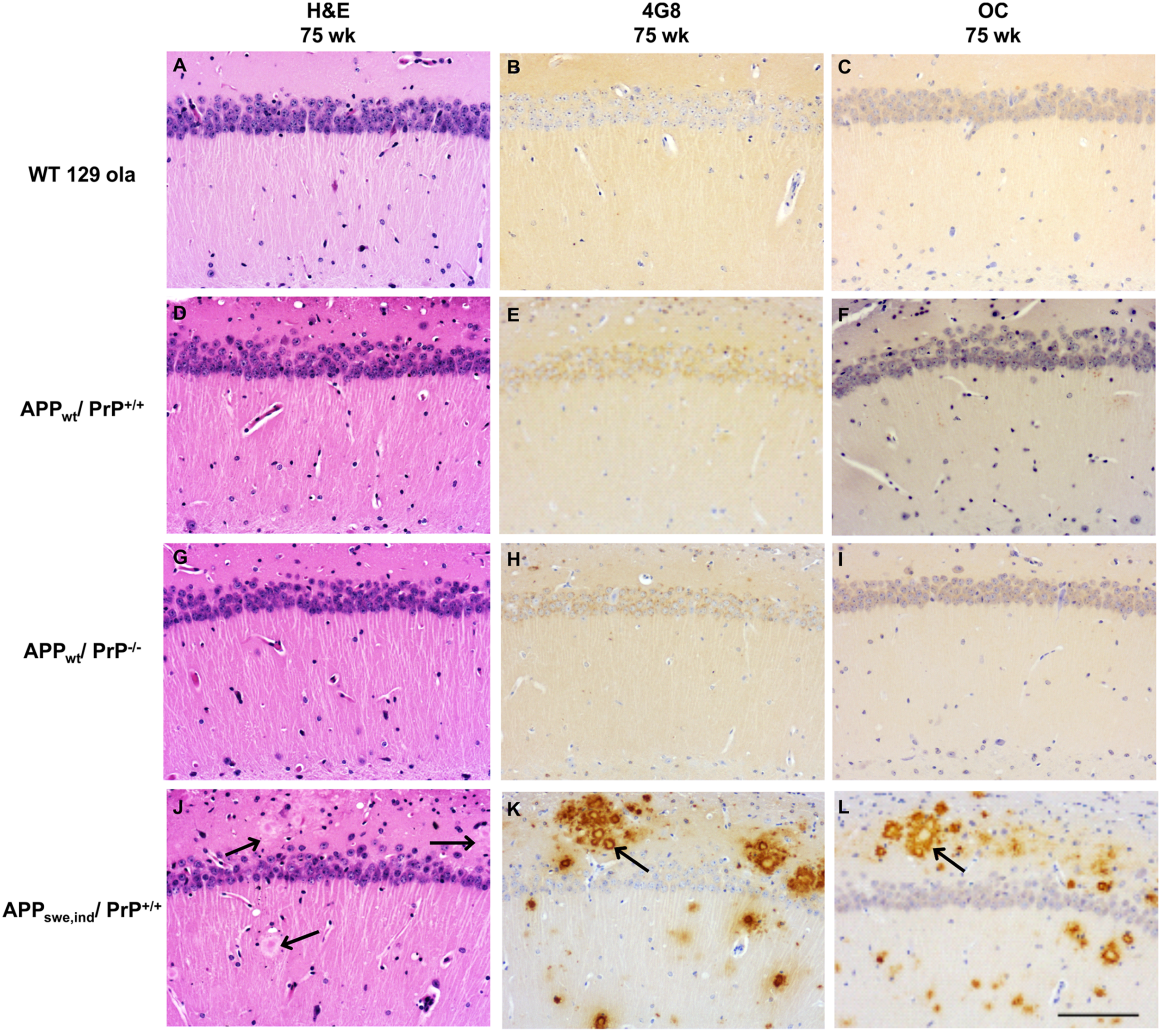
Ablation of PrP^C^ does not affect Aβ deposition in APP_WT_ mice. Histopathologic analysis of the hippocampus of hematoxylin-eosin stained sections of WT, APP_WT_/PrP^++^ and APP_WT_/PrP^--^ mice show absence of obvious alterations in the pyramidal cell layer, stratum oriens and lacunosum-molecular (A, D and G). No Aβ deposits are seen in sections probed with antibody 4G8 (B, E and H) or antibody OC (C, F and I). In contrast, APP_Swe,Ind_/PrP^+/+^ mice show plaques (arrows) in sections stained with hematoxylin-eosin (J) and abundant Aβ-reactive deposits in sections probed with antibody 4G8 (K) and OC (L). All images x20 magnification and scale bar = 100μm.

**Fig 3:**
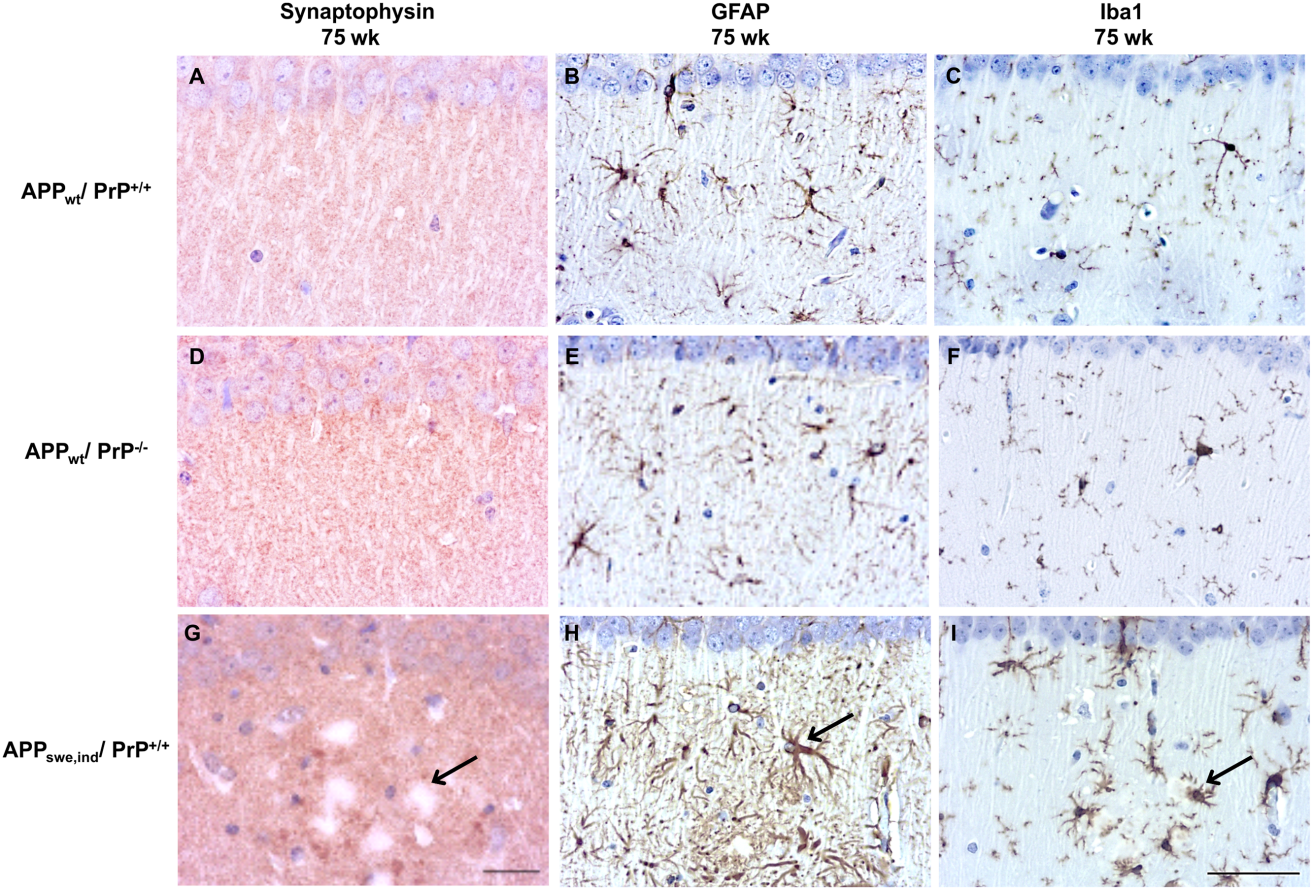
Ablation of PrP^C^ does not affect synaptophysin immunoreactivity and does not induce gliosis in APP_WT_ mice. APP_WT_/PrP^+/+^ and APP_WT_/PrP^-/-^ mice show retention of the pre-synaptic marker synaptophysin (A, D) while APP_Swe.Ind_/PrP^+/+^ mice reveal disorganization of synaptophysin immunoreactivity (arrow) (G). Astrogliosis and microgliosis are observed in 75 week old APP_Swe.Ind_/PrP^+/+^ mice, (arrows) (H, I) but not in mice expressing APP_WT_/PrP^+/+^ and APP_WT_/PrP^-/-^ (B, C, E, F). Section were probed with anti-synaptophysin (A, D, G), anti-GFAP (B, E, H), and anti-Iba1 (C, F, I) antibodies. Figures A,D,G (synaptophysin) x60 magnification scale bar = 20μm, figures B,E,H (GFAP) and C,F,I (Iba1) x40 magnification scale bar = 50μm.

**Fig 4:**
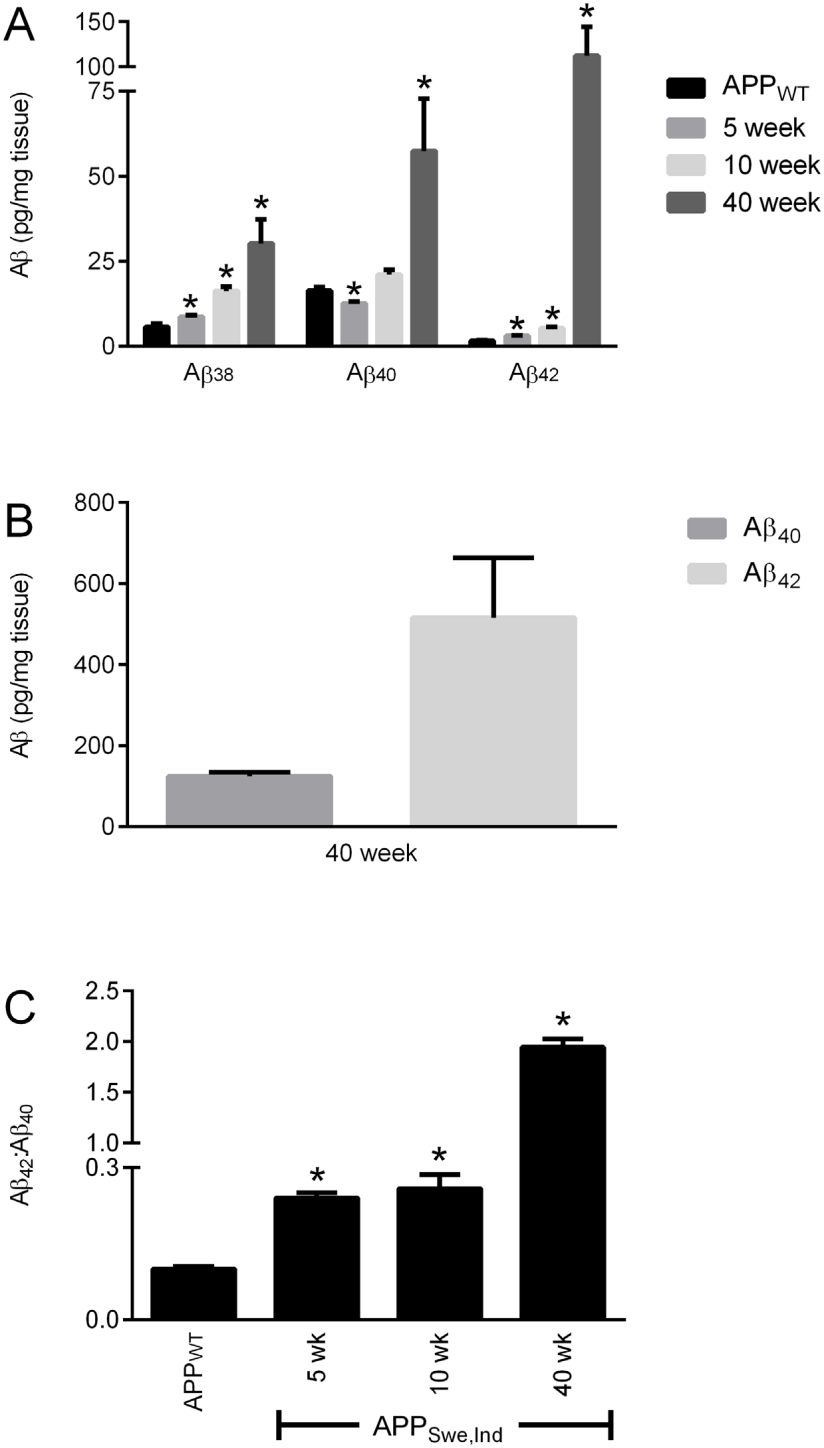
Comparison of Aβ levels in APP_WT_ and APP_Swe.Ind_ mice. Brain hemispheres from 75 week old APP_WT_/PrP^+/+^ and from 5, 10 and 40 week old APP_Swe,Ind_/PrP^+/+^ mice were subjected to two-step homogenisation. (A) Soluble (SDS extracted) and (B) insoluble (formic acid extracted) Aβ_38,_ Aβ_40_ and Aβ_42_ detected by MSD ELISA and (C) the ratio of Aβ_42:_Aβ_40_. Data shown as mean ± SEM (n = 3–6), * p<0.05 compared to the 75 week old APP_WT_/PrP^+/+^ group.

To investigate whether lack of PrP^C^ in the APP_WT_/PrP^-/-^ mice affected the deposition of Aβ in the brain, histopathological analysis of the hippocampus was carried out at 32 weeks of age and compared to APP_WT_/PrP^+/+^ mice. APP_Swe,Ind_/PrP^+/+^ mice, which are known to accumulate limited Aβ-positive plaques at this age, were also analysed [9,21]. APP_WT_/PrP^+/+^ and APP_WT_/PrP^-/-^ mice at 32 weeks of age showed no obvious neuropathologic alterations (data not shown). To explore whether histopathologic alterations were detected in older animals, we also examined mice at 75 weeks of age. Histopathological analysis of WT, APP_WT_/PrP^+/+^ and APP_WT_/PrP^-/-^ mice at 75 weeks of age showed similar histopathologic features; i.e. absence of obvious neuronal loss or other obvious alterations in the hippocampus proper or dentate gyrus (Fig 2A, 2D and 2G) or 4G8 positive Aβ plaque formation (Fig 2B, 2E and 2H) in these mice. We investigated whether the ablation of PrP^C^ affected the level of Aβ fibrils and fibrillar oligomers by using the OC antibody [25]. We observed that WT, APP_WT_/PrP^+/+^ and APP_WT_/PrP^-/-^ mice did not accumulate OC immunopositive deposits as shown by immunohistochemistry (Fig 2C, 2F and 2I). As expected, in the APP_Swe,Ind_/PrP^+/+^ mice at 75 weeks of age there was disruption of the hippocampal structure with accumulation of 4G8 and OC positive Aβ deposition forming numerous uni and multicentric plaques (Fig 2J, 2K and 2L), as seen previously [9]. Synapses are particularly vulnerable to the toxic effect of protein oligomers. Thus, we performed immunostaining for the detection of the presynaptic protein synaptophysin and observed disruption of immunoreactivity in 75 week old APP_Swe,Ind_/PrP^+/+^ mice but not in APP_WT_/PrP^+/+^ or APP_WT_/PrP^-/-^ mice (Fig 3A, 3D and 3G). Glial markers (GFAP and Iba1) showed similar intensity and pattern of reactivity in APP_WT_/PrP^+/+^ and APP_WT_/PrP^-/-^ mice (Fig 3B, 3C, 3E and 3F). In contrast, gliosis was observed particularly in the vicinity of amyloid deposits in APP_Swe,Ind_/PrP^+/+^ mice (Fig 3H and 3I). Thus, we did not detect any effect of PrP^C^ ablation on Aβ deposition using immunohistochemistry.

A comparison of the Aβ levels between the APP_WT_/PrP^+/+^ mice at 75 weeks of age with APP_Swe,Ind_/PrP^+/+^ mice at 5 and 10 weeks (before significant Aβ deposition occurs) and at 40 weeks (after Aβ deposition in plaques appears) revealed that at 75 weeks of age the amount of Aβ peptides in the soluble (SDS extracted) fraction of the APP_WT_/PrP^+/+^ mice was comparable to the amount of each peptide in the 5 week old APP_Swe,Ind_/PrP^+/+^ mice (Fig 4A). Although Aβ_40_ and Aβ_42_ were readily detectable in the insoluble (formic acid extracted) fraction from the APP_Swe,Ind_/PrP^+/+^ mice (Fig 4B), no Aβ was detected in the insoluble fraction from the APP_WT_/PrP^+/+^ mice. The soluble Aβ_42_:Aβ_40_ ratio increased with age in both APP_WT_/PrP^+/+^ and APP_Swe,Ind_/PrP^+/+^ mice (Fig 4C) but was significantly higher in the APP_Swe,Ind_/PrP^+/+^ mice than in the APP_WT_/PrP^+/+^ mice consistent with the presence of the Indiana mutation increasing production of Aβ_42_ as observed previously [21]. Even at 75 weeks of age the amount of Aβ_42_ in the APP_WT_/PrP^+/+^ mice is significantly lower than that in the APP_Swe,Ind_/PrP^+/+^ mice at 5, 10 and 40 weeks of age (Fig 4A). Thus in the APP_WT_/PrP^+/+^ mice even at 75 weeks of age the low level of Aβ_42_ is likely below the threshold required for deposition to occur.

## Conclusion

In cellular models PrP^C^ differentially affected the activity of the β-secretase BACE1 towards APP_WT_ and APP_Swe_, inhibiting the processing of the former but having no effect on the cleavage of the latter [8,9]. Thus we undertook this study in order to investigate the effect of genetic ablation of PrP^C^ on human APP_WT_ processing *in vivo*. In contrast to the cellular models, lack of PrP^C^ had no effect on the α- and β-secretase proteolysis of APP or on the amount of Aβ peptides in the brains of mice expressing human APP_WT_. Also we did not detect any Aβ deposition as shown by immunohistochemistry in the mice lacking PrP^C^ as compared to those with a normal level of the protein. In the human brain the amount of PrP^C^ inversely correlated with BACE1 activity, soluble and insoluble Aβ and Braak stage [11], consistent with levels of PrP^C^ affecting APP processing and Aβ production. However, we could find no evidence that loss of PrP^C^ affected APP proteolysis, Aβ levels or plaque pathology *in vivo* in transgenic mice expressing human APP_WT_. Whether this reflects a difference in the role of PrP^C^ in regulating APP processing and Aβ production between this transgenic mouse model and the situation in the human brain will require further study.

## Abbreviations

Aβ: amyloid-β
AD: Alzheimer’s disease
APP: amyloid precursor protein
BACE1: β-site APP cleaving enzyme-1
PrP^C^: prion protein
